# Negative Schizotypy Associated With Weaker Intersubject Correlation in Dynamic Functional Connectivity During Empathic Accuracy Task

**DOI:** 10.1093/schbul/sbad182

**Published:** 2025-03-04

**Authors:** Ding-ding Hu, Xiao-dong Guo, Simon S Y Lui, Yi Wang, Raymond C K Chan

**Affiliations:** Neuropsychology and Applied Cognitive Neuroscience Laboratory, CAS Key Laboratory of Mental Health, Institute of Psychology, Chinese Academy of Sciences, Beijing, China; Department of Psychology, University of Chinese Academy of Sciences, Beijing, China; Neuropsychology and Applied Cognitive Neuroscience Laboratory, CAS Key Laboratory of Mental Health, Institute of Psychology, Chinese Academy of Sciences, Beijing, China; Department of Psychology, University of Chinese Academy of Sciences, Beijing, China; Department of Psychiatry, School of Clinical Medicine, The University of Hong Kong, Hong Kong Special Administrative Region, China; Neuropsychology and Applied Cognitive Neuroscience Laboratory, CAS Key Laboratory of Mental Health, Institute of Psychology, Chinese Academy of Sciences, Beijing, China; Department of Psychology, University of Chinese Academy of Sciences, Beijing, China; Neuropsychology and Applied Cognitive Neuroscience Laboratory, CAS Key Laboratory of Mental Health, Institute of Psychology, Chinese Academy of Sciences, Beijing, China; Department of Psychology, University of Chinese Academy of Sciences, Beijing, China

**Keywords:** social anhedonia, perceptual aberration, empathy, neural synchronization, mediation effect

## Abstract

**Background and Hypothesis:**

Previous studies on Empathic Accuracy Task (EAT) suggested patients with schizophrenia exhibited altered brain activations in the precuneus, middle frontal gyrus, and thalamus. However, it remains unclear whether individuals with schizotypy would exhibit similar alterations of brain activations associated with EAT. This study aimed to examine the relationships between schizotypy and intersubject correlation (ISC) during EAT.

**Study Design:**

Forty-seven college students undertook the Chinese version of EAT in a 3T MRI scanner. The Chapman Social Anhedonia Scale (CSAS) and Perceptual Aberration Scale (PAS) were used to capture negative and positive schizotypy, respectively. We adopted GLM analysis, ISC analyses of brain activation, and dynamic functional connectivity during EAT to examine its association with dimensional schizotypy and self-report empathy.

**Study Results:**

Regardless of schizotypy scores, brain activations in the middle occipital cortex, precuneus, lingual gyrus, paracentral gyrus, and anterior cingulate cortex (ACC) were associated with participants’ empathic accuracy, while strong ISC of brain activations were found in bilateral superior temporal gyri (STG). Negative schizotypy was associated with ISC of brain activation in the precentral gyrus and dynamic connectivity between the STG and ACC, both of which further mediated the associations between negative schizotypy and self-report affective empathy.

**Conclusions:**

These preliminary findings suggest that weaker intersubject synchronization of brain activation in the precentral gyrus and dynamic connectivity between the STG and ACC is related to negative schizotypy. Our findings may shed light on the underlying neural mechanisms of impaired social cognition in patients with schizophrenia spectrum disorder.

## Introduction

Social cognitive impairment is an important feature of schizophrenia, and is closely related to dysfunction in everyday life.^1^  *Empathy* is an important aspect of social cognition, and is defined as the ability to recognize, understand, and feel others’ emotional states.^2^ Current literature suggests that empathy comprises the cognitive domain (i.e., the ability to understand and make inferences of others’ emotional and mental states) and affective domain (i.e., the ability to share emotions with others).^3,4^ Although empirical evidence shows that schizophrenia patients exhibit impairments in both cognitive and affective empathy, its neural mechanisms and relationship with clinical symptoms remain unclear.^5,6^ On the other hand, *schizotypy* is a latent personality structure that confers liability to schizophrenia and related psychosis.^7^ Studying schizotypy provides an opportunity to investigate the neural mechanisms associated with schizophrenia, while avoiding common confounding factors in schizophrenia patients, such as antipsychotic medication and institutionalization.^8^ The extant literature supported a three-dimension structure of schizotypy, including the positive (e.g., perceptual aberration, idea of references), the negative (e.g., no close friend, constricted affect), and the disorganized (e.g., odd behavior or speech) dimensions.^9^ As a key aspect of the negative schizotypy, social anhedonia refers to diminished pleasure experiences from social events.^10^ Negative schizotypy is consistently associated with cognitive and affective empathy impairments,^11–14^ yet very few studies had been conducted to investigate the neural mechanisms for cognitive and affective empathy in people with negative schizotypy.

 Empathic Accuracy (EA), the degree to which an individual can accurately understand others’ thoughts and feelings,^15,16^ is essential for social interaction. EA can be measured using paradigms such as the *Empathic Accuracy Task (EAT)* developed by Zaki, Bolger, and Ochsner^17^. In the EAT, participants watch a series of video clips in which a target is talking about an autobiographical event, and are asked to continuously evaluate the target’s emotional valence during video playback^17^ or to report the target’s overall emotion after the video clip finishes.^18^ Then, a participant’s EA could be estimated by either calculating the *correlation coefficient* between his/her ratings and the target’s ratings,^17^ or the *difference* between overall emotions rated by participants and targets.^18^ EAT paradigms have been widely used to measure empathy in clinical samples, and achieved high ecological validity and reliability (see a review^19^). Using EAT, evidence has consistently found lower EA in schizophrenia patients relative to controls.^20–23^ Our preliminary analysis has applied EAT in people with high levels of schizotypy, and found lower EA scores.^24^

Adopting the EAT, functional MRI studies have shown increased brain activation in the medial prefrontal cortex (mPFC), precuneus, and posterior cingulate in healthy people.^25–27^ Moreover, brain activations in the superior temporal sulcus (STS), the temporo-parietal junction (TPJ), the inferior parietal cortex (IPL), and the precuneus were found to positively predict EA scores.^25,26,28^ To date, however, only two previous studies utilized EAT to examine brain activation and functional connectivity in patients with schizophrenia spectrum disorders. One study found diminished brain activations during EAT in the left precuneus, left middle frontal gyrus, and bilateral thalamus in schizophrenia patients compared with controls.^29^ Another study identified three data-driven networks during EAT, yet did not find any significant difference in network connectivity between patients with schizophrenia spectrum disorders and controls.^30^

*Intersubject correlation*  *(ISC) analysis* is a useful method to measure the consistency across individuals on brain activation^31,32^ or functional connectivity^33–35^ when they are engaged by the same naturalistic and dynamic stimuli. This method can better identify neural activity patterns underlying complex cognitive processes.^36,37^ The ISC analysis has been used to examine “neural synchronization” of brain activation in healthy people,^31,38,39^ and found strong ISC of widespread regions involved in sensory and perceptual processing as well as higher-level cognitive processing (e.g., the precuneus, TPJ, ACC, and mPFC). On the other hand, schizophrenia patients showed lower within-group ISC in the TPJ, mPFC, precuneus, and dorsolateral prefrontal cortex (dlPFC) relative to controls,^40,41^ and altered intersubject functional connectivity of the TPJ during task-based fMRI.^42^ In people with schizotypy, a recent study suggested negative correlations between the ISC of brain activation in the middle/inferior frontal gyrus during a movie-watching task and the ratings of the Oxford-Liverpool Inventory of Feelings and Experiences (a measure for schizotypal traits).^43^ Moreover, people with high levels of paranoia (resembling those with positive schizotypy) exhibited higher ISC of brain activations in the left temporal pole, precuneus, and right mPFC, relative to their counterparts with low levels of paranoia.^44^ Together, these findings suggested that dimensional schizotypy may manifest distinct associations with the ISC of brain activation. However, the underlying association between schizotypy and ISC of functional connectivity remains unclear. The ISC analysis approach can allow us to examine the underlying neural synchronization when participants with schizotypy were performing the EAT.

This study aimed to examine the relationships between schizotypy and ISC during EAT. First, we investigated the brain activation related to *emotional distance* using the General Liner Models (GLM) as well as the ISC of brain activation during video-watching phase. Second, we examined the ISC of dynamic connectivity (ISDC) based on the seeds we found from the GLM and ISC analyses. Then, we tapped into the associations between schizotypy and ISC in brain activation and dynamic connectivity using multiple regression analysis. Finally, the correspondence values of those clusters related to schizotypy were extracted, for estimating their correlations with self-report cognitive and affective empathy. We hypothesized that (1) widespread ISC of brain activation in the audio-visual processing areas (e.g., superior and middle temporal gyri) and social brain network (e.g., TPJ, precuneus, mPFC, insula, IPL); (2) significant ISDC between brain regions within these networks; and (3) negative correlations between schizotypy and the ISC as well as the ISDC during EAT would be observed.

## Methods

### Sample

Fifty-three college students were recruited through online advertisements in mainland China. The inclusion criteria included (1) right-handedness, (2) normal or corrected-to-normal vision, and (3) absence of any neurological or psychiatric disorders. After online screening, we excluded 3 participants due to psychiatric condition (*n* = 1) and left handedness (*n* = 2). Among 50 participants who underwent fMRI brain scan, three were excluded due to excessive head motion (maximum FD greater than 1.5 mm or 1.5 degrees)^45^. The final sample comprised 47 participants (27 females, mean age = 23.40 years, SD = 2.46, mean years of education = 16.19, SD = 1.87). This study was approved by the Ethics Committee of the Institute of Psychology, the Chinese Academy of Sciences (No. H20045). All participants provided written consent form before the experiment began. They received monetary remuneration (approximately US $20) after completion of this study.

### Measurement of Schizotypal Features and Self-report Empathy

The 40-item self-report Chapman Social Anhedonia Scale (CSAS)^10^ and the 35-item Perceptual Aberration Scale (PAS)^46^ were used to measure negative and positive dimensions of schizotypy, respectively. The CSAS captured the diminished pleasure from social activities, and the PAS measured the abnormal perceptual experiences. The Chinese version of both scales demonstrated good validity and reliability.^47,48^

The 31-item self-report Questionnaire of Cognitive and Affective Empathy (QCAE)^3^ was used to measure cognitive and affective components of empathy. The Chinese version of QCAE showed good reliability and validity in both healthy population^49^ and clinical sample with psychiatric disorders.^50^

### Experimental Task in the Scanner

During the fMRI scanning, participants completed the Chinese version of EAT^51^ in two runs. Each run started with a 23-second fixation (excluded from the analysis) for noise reduction and then played video clips (lasting 2–4 min) in which showed a target describing his/her autobiographical emotional events with a 12-second interval in between. Using a horizontal 9-point Likert scale (1 = very negative, 5 = neutral, 9 = very positive), participants continuously rated the emotional valence of the target as they were watching the video clips, by moving the two buttons of the response box (1 = move to left; 2 = move to right) with the right hand. Eight video clips were presented sequentially in two functional runs (with 2 positive and 2 negative in each run) in a fixed order. The first run lasted for 685 seconds, and the second run lasted for 695 seconds. The video clips were presented using E-prime 3.0 with high-fidelity MR-compatible earphones (OptoACTIVE) to minimize the noises inside the scanner, and to ensure optimal audibility of the narrative contents. The Chinese version of EAT has been detailed elsewhere.^24,51^

For each video clip, participants’ continuous ratings were interpolated and averaged every 2 seconds. *EA* was estimated using Spearman’s correlation between the target’s and each participant’s ratings. Furthermore, we calculated the absolute difference between the target’s and each participant’s ratings in every 2 seconds to generate *Emotional Distance*, which reflects participants’ ability of accurately tracking the target’s emotional states.

### The fMRI Scanning and Preprocessing

A 3T GE Discovery MR750 scanner and an 8-channel head-coil were used for MRI data acquisition. Details on scan parameters and fMRI preprocessing are shown in supplementary materials.

### GLM Analyses

A general linear model was built in the first-level analysis for each participant as a block design, with the time series of *emotional distance* scores entered as the regressor of interest, and the six head motion parameters as nuisance regressors. All regressors were convolved with the canonical hemodynamic response function (HRF) with a high-pass filter of 128 seconds. To evaluate participants’ brain activation when performing EAT, as well as the specific changes in BOLD signals related to *emotional distance*, three first-level contrasts were estimated separately for (1) video-watching, (2) positive association with *emotional distance*, and (3) negative association with *emotional distance*. In the second-level analysis, one-sample *t*-tests of all first-level contrasts were conducted within a gray matter mask of the cerebrum. Significant thresholds were set at voxel-level uncorrected *P* < .001 and cluster-level family-wise error (FWE) corrected *P* < .05.^28^

### ISC Analyses for Brain Activation

Potential effects of head motion estimated by Friston’s 24-parameter^52^ were regressed out to create residual images of each individual participant for ISC analyses. Following the pipeline of Di and Biswal,^33^ the BOLD time series of each voxel during video-watching phase for each participant were extracted and concatenated in a fixed order, within a whole-brain gray matter mask. Then, the leave-one-out ISC approach^32^ was applied. Specifically, we calculated the correlation coefficients between the BOLD time series of one participant and the average time series of the remaining *N*–1 participants at each voxel, resulting in the map for ISC in brain activation for each individual participant. Group-level averaged ISC map in brain activation was calculated using the fisher-*z* transformed *r* maps of all participants. To provide information of real effect size, group-level *z* map was transformed back to *r* map.^33^ Taking reference to the previous study,^33^ we adopted a relatively high threshold of *r* > 0.45 to identify regions with strong ISC. In addition, a relatively lenient threshold of *r* > 0.15^53^ was also adopted to reveal a general pattern of ISC during EAT.

### ISC Analyses for Dynamic Connectivity (ISDC)

Based on the results from above mentioned GLM analyses and ISC analyses in brain activation, we defined the regions of interest for ISDC analyses to identify ISC in dynamic functional connectivity. Sliding window technique was applied with window length of 60 seconds and time step of 4 seconds^33^ to calculate the dynamic connectivity of each seed with every other voxel within a whole-brain gray matter mask. Then leave-one-out ISC on seed-based dynamic connectivity was calculated and fisher-*z* transformed for each participant. Group-level one-sample *t*-test was then performed with a significant threshold of voxel-level uncorrected *P* < .001 and cluster-level FWE corrected *P* < .05.

### Neural Correlates of Dimensional Schizotypy

To examine the associations between schizotypy and brain activation, ISC in brain activation, as well as seed-based ISDC, multiple regression analyses were performed with the CSAS or PAS scores as covariate of interest, while controlling for age and sex at birth. Since the clusters would be extracted for subsequent analyses, we selected a more lenient significance threshold of voxel-level uncorrected *P* < .005 and cluster-level FWE corrected *P* < .05.^54,55^

### Correlations and Mediation Effect Analysis

Corresponding values of the significant clusters associated with schizotypy were extracted for subsequent correlation analyses with the QCAE. *Bonferroni* corrections were used to control for multiple comparisons. If significant correlation with scores of the QCAE was observed, we would examine the mediation effect^56^ of neural correlates on the associations between schizotypy and self-report empathy. The mediation effect was examined with 5000 times bootstrapping using the SPSS PROCESS macro (version 2.16, model 4).^57^

## Results

### Associations Between Schizotypy and Empathy

Demographic information and descriptive statistics are shown in table 1. The CSAS scores showed significant negative correlations with cognitive (*r* = −0.41, *P* = .005) and affective (*r* = −0.45, *P* = .001) dimensions of the QCAE, while the PAS scores did not show any significant correlation with the QCAE. All of the correlations between schizotypy and EAT performances failed to reach statistical significance (*P*s > .05).

**Table 1.** Demographics, Self-report Scales, and EAT Performance

	Entire sample (*n* = 47)			
	Mean	SD	Range	
			Min	Max
Age (years)	23.40	2.46	18	28
Years of Education	16.19	1.87	12	20
Gender (Male:Female)	20:27			
CSAS	10.64	8.92	0	34
PAS	3.85	3.24	0	17
QCAE				
Cognitive Empathy	59.17	8.14	39	74
Perspective taking	31.74	6.07	16	43
Online simulation	27.43	2.80	22	33
Affective empathy	30.60	4.30	22	38
Emotion contagion	10.79	2.26	4	16
Proximal responsivity	8.38	1.39	6	12
Peripheral responsivity	11.43	2.24	6	16
EAT performance				
Fisher-*z* transformed EA	1.11	0.31	0.13	1.64
Emotional distance	0.97	0.28	0.48	1.67

*Note*: EAT, Empathic Accuracy Task; CSAS, Chapman Social Anhedonia Scale; PAS, Perceptual Aberration Scale; QCAE, Questionnaire of Cognitive and Affective Empathy; EA, empathic accuracy.

### Brain Activation Associated With Emotional Distance During the EAT

**Fig. 1.**
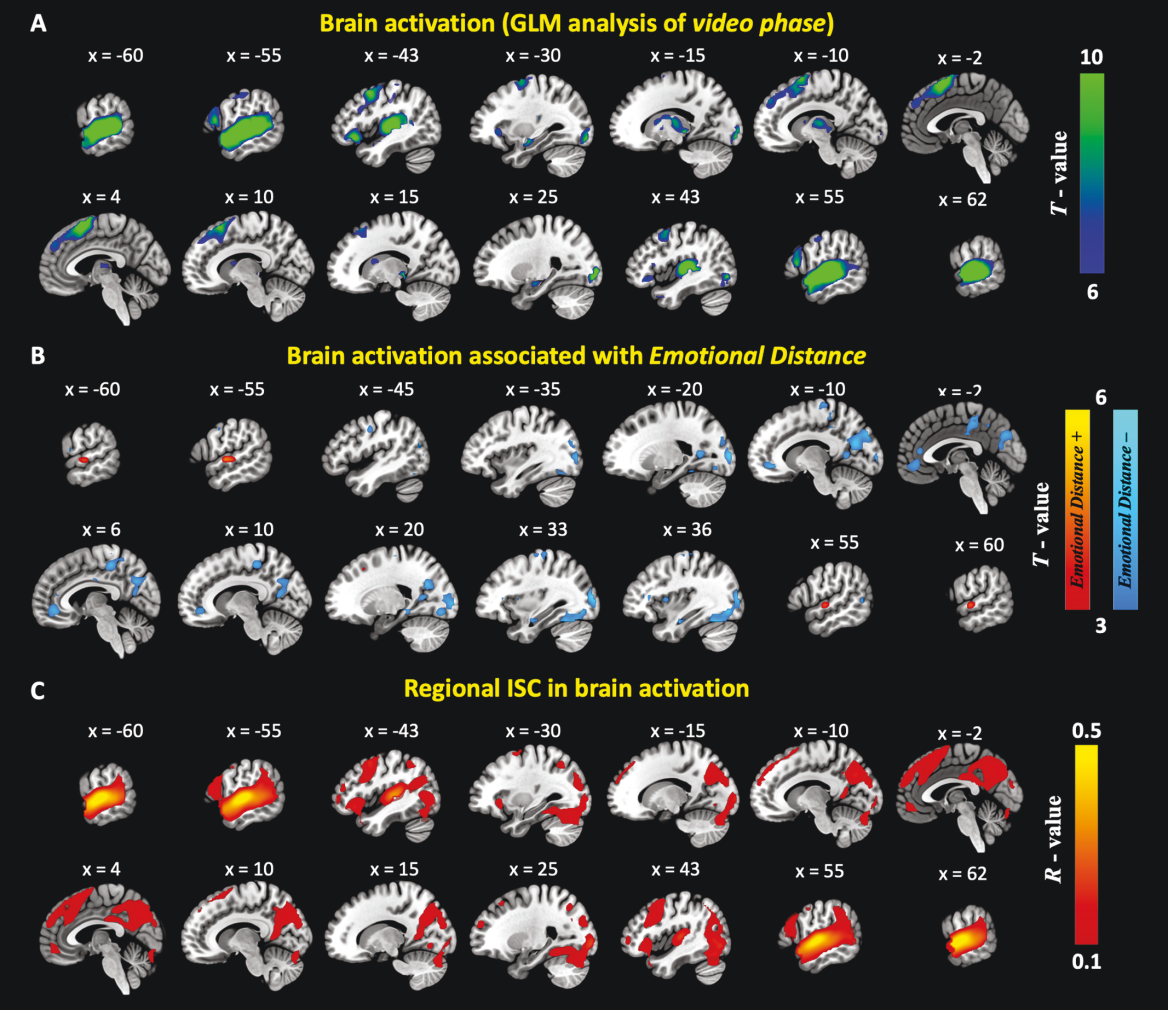
Brain activations during the video-watching phase of the Empathic Accuracy Task (panel A) and brain activations associated with *Emotional Distance* (panel B) using GLM analyses. One-sample *t*-tests were conducted with significant threshold set at voxel-level uncorrected *P* < .001 and cluster-level family-wise error (FWE) corrected *P* < .05. Panel C: group-level *r*-map of the intersubject correlation (ISC) in brain activations during EAT with a threshold of *r *> 0.1.

One-sample *t*-tests showed that *Emotional Distance* was negatively associated with neural activities in bilateral middle occipital gyrus (MOG), left precuneus (PCU), right lingual gyrus (LG), right paracentral gyrus (PaCG), and right anterior cingulate cortex (ACC) (see figure 1 and table 2), while no clusters showing significant positive correlation with emotional distance were found.

**Table 2.** Significant Clusters of Brain Activations Associated With Emotional Distance

Brain region	L/R	BA	Cluster Size	MNI coordinates			*t*	*P*
				x	y	z		
Middle occipital gyrus	L	18	115	−21	−96	6	5.29	.018
Middle occipital gyrus	R	18	141	33	−87	9	5.22	.008
Precuneus	L	31	381	−9	−69	21	4.79	<.001
Lingual gyrus	R	18	169	36	−72	−9	4.68	.004
Paracentral lobule	R	7	138	9	−30	51	4.48	.009
Anterior cingulate cortex	R	24	107	6	42	−6	4.13	.023

*Note*: Significant threshold was set at voxel-level uncorrected *P* < .001 and cluster-level family-wise error (FWE) corrected *P* < .05. L, left; R, right; BA, Brodmann Areas; MNI, Montreal Neurological Institute. These clusters were defined as regions of interest for further intersubject dynamic connectivity (ISDC) analyses.

### ISC in Brain Activation During the EAT

Applying a threshold of *r* > 0.45, strong ISC in brain activation during EAT was found in bilateral superior temporal gyri (STG). In addition, applying a threshold of *r* > 0.15, we found relatively high ISC in bilateral precuneus, dlPFC, LG, insula, TPJ, left inferior frontal gyrus (IFG), and right dorsomedial prefrontal cortex (dmPFC) (see figure 1).

### ISC in Dynamic Functional Connectivity During the EAT

**Fig. 2.**
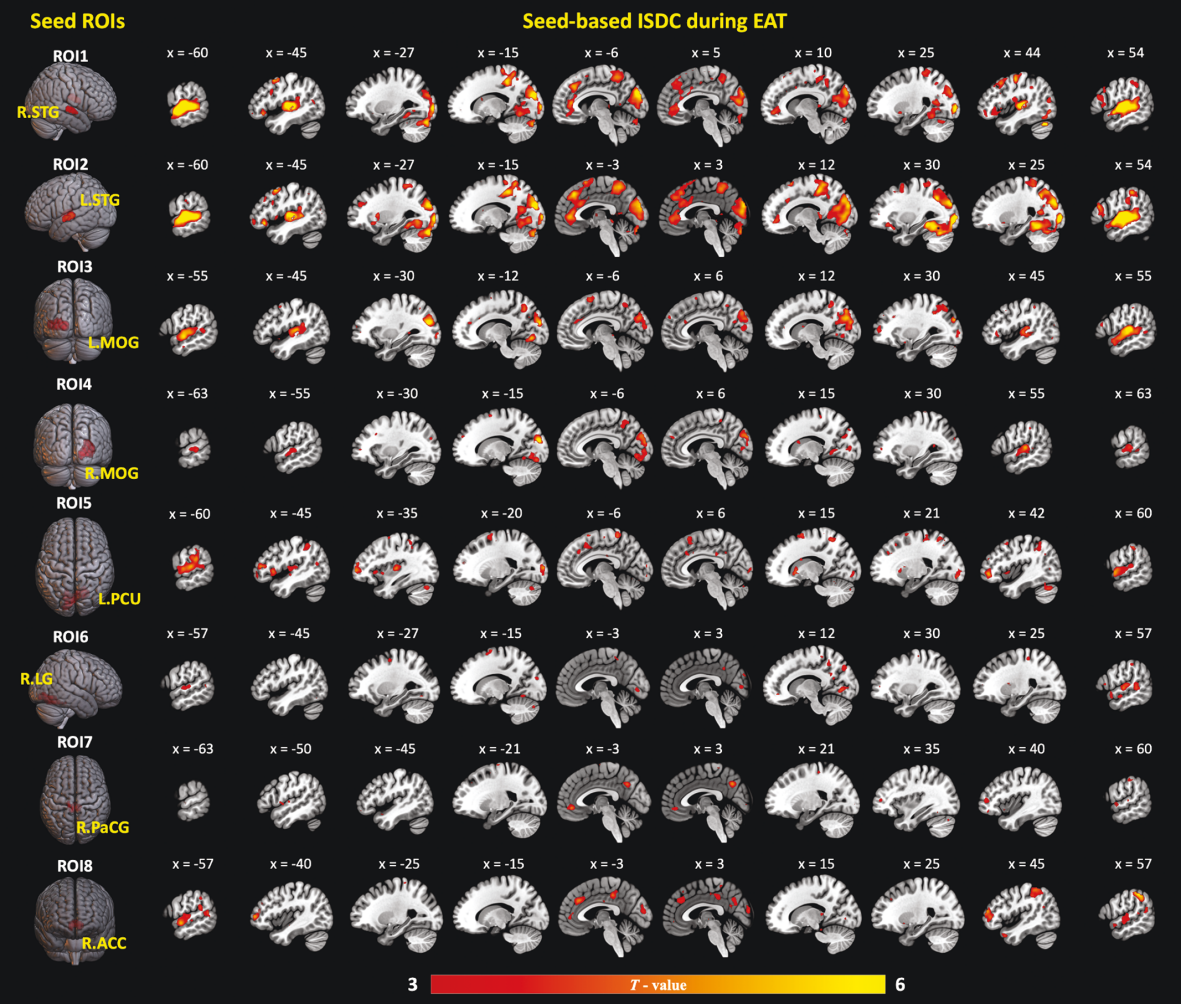
Intersubject correlation of dynamic functional connectivity (ISDC) for regions of interest (ROI) defined by results of the ISC analyses (ROI1–ROI2) and GLM analyses (ROI3–ROI8) in brain activations. One-sample *t*-tests were conducted with significant threshold set at voxel-level uncorrected *P* < .001 and cluster-level family-wise error (FWE) corrected *P* < .05. EAT, Empathic Accuracy Task; R.STG, right superior temporal gyrus; L.STG, left superior temporal gyrus; L.MOG, left middle occipital gyrus; R.MOG, right middle occipital gyrus; L.PCU, left precuneus; R.LG, right lingual gyrus; R.PaCG, right paracentral gyrus; R.ACC, right anterior cingulate cortex.

As shown in figure 2, we found similar patterns for the ISDC of bilateral STG (ROI1 and ROI2), indicating significant ISC in dynamic connectivity between the STG and occipital lobe, medial frontal gyrus, ACC, middle frontal gyrus, precentral gyrus, IFG, superior frontal gyrus (SFG), and insula. Bilateral MOG (ROI3 and ROI4) exhibited significant ISDC with the STG and precuneus. Left PCU (ROI5) showed significant ISDC with bilateral STG, IPL, right orbital frontal gyrus, and right angular gyrus (AG), as well as left dlPFC and dmPFC. Right LG (ROI6) showed significant ISDC with right STG and MTG. Right PaCG (ROI7) exhibited significant ISDC with right precuneus and left ACC. Right ACC (ROI8) showed significant ISDC with bilateral STG, dlPFC, right IPL, left middle, and posterior cingulate cortex. Details are shown in supplementary materials (table S3).

### Neural Correlates of Dimensional Schizotypy

We found negative correlations between the CSAS scores and ISC of brain activation in the left precentral gyrus (MNI coordinates: [−36, −30, 66], *t* = 5.10, *k* = 174, *P* = .007). In addition, the ISDC between left STG (ROI2) and right ACC, extending to dmPFC (MNI coordinates: [15, 39, 18], *t* = 4.90, *k* = 280, *P* < .001) was negatively correlated with the CSAS scores. We also found that the PAS scores were negatively correlated with the ISDC between right PaCG (ROI7) and right LG (MNI coordinates: [9, −72, −12], *t* = 4.45, *k* = 123, *P* = .003). No significant correlations between scores on the CSAS or PAS and brain activities associated with *emotional distance* was found.

### Correlation and Mediation Effect Analysis

**Fig. 3.**
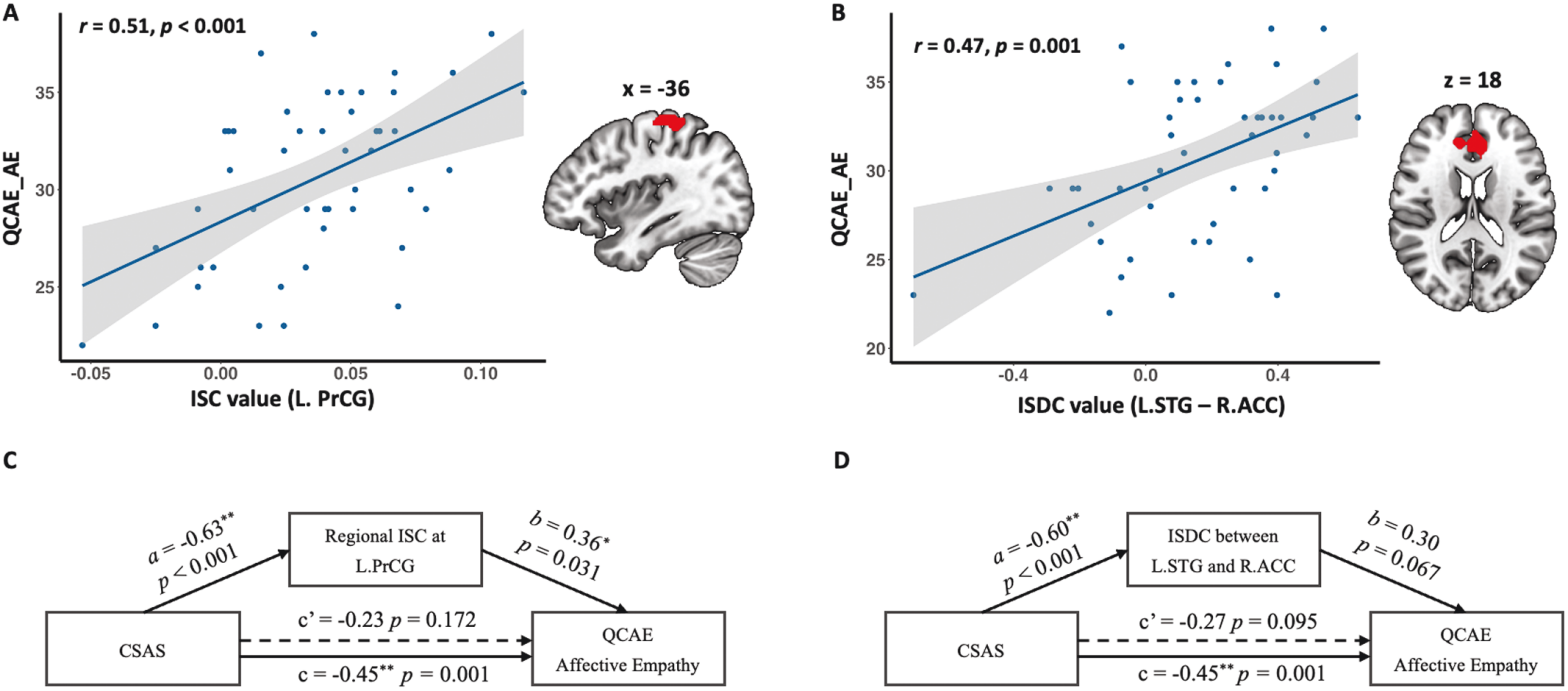
Pearson correlation between affective empathy dimensional scores of the QCAE and the ISC of brain activation in the L.PrCG (panel A), and the ISDC between the L.STG and R.ACC (panel B). Results of the mediation effect analyses of the ISC of brain activation in the L.PrCG (panel C), as well as the ISDC between the L.STG and R.ACC (panel D) on the association between negative schizotypy and affective empathy. ISC, intersubject correlation in brain activation; ISDC, intersubject correlation in dynamic connectivity; QCAE, Questionnaire of Cognitive and Affective Empathy; AE, Affective Empathy; CSAS, Chapman Social Anhedonia Scale; L.PrCG, left precentral gyrus; L.STG, left superior temporal gyrus; R.ACC, right anterior cingulate cortex.

Among those clusters associated with schizotypy, we found significant correlations between scores on affective empathy of the QCAE and the ISC in left precentral gyrus (*r* = 0.51, *P* < .001), as well as the ISDC between left STG and right ACC (*r* = 0.47, *P* = .001) after *Bonferroni* corrections (figure 3). The positive correlation between cognitive empathy of the QCAE and the ISDC between left STG and ACC (*r* = 0.29, *P* = .047) failed to survive *Bonferroni* correction. No other significant correlations were found between self-report empathy and neural correlates of schizotypy.

Mediation effect analysis showed that the ISC in left precentral gyrus exhibited an indirect effect of the CSAS on affective empathy of the QCAE (a*b = −0.23, CI = [−0.482, −0.011]), resulting in a full mediation effect (*cʹ*-path), and explained 50% of the variance. In addition, the ISDC between left STG and ACC exhibited an indirect effect of the CSAS on affective empathy of the QCAE (a*b = −0.18, CI = [−0.403, −0.010]), also resulting in a full mediation effect (*cʹ*-path), and explained 40% of the variance (figure 3).

## Discussion

Our findings showed that brain activities in bilateral MOG, left precuneus, right LG, right PaCG, and right ACC were significantly related to emotional distance. ISC analyses suggested strong neural synchronization across participants in brain regions involved in the empathy/mentalizing network (such as the STG, TPJ, precuneus, and dmPFC) and the mirror-neuron network (such as the IFG and IPL). More importantly, negative schizotypy was negatively correlated with the ISC in left precentral gyrus, as well as the ISDC between left STG and right ACC, both of which served as mediators on the relationship between social anhedonia and affective empathy. In addition, positive schizotypy was negatively associated with the ISDC between right paracentral gyrus and right lingual gyrus.

Similar to previous fMRI studies using EAT,^26,27^ we found that accurately tracking of other’s emotion relates to increased brain activation in the MOG, precuneus, LG, PaCG, and ACC. Increased brain activation of these regions have been observed during mentalizing tasks.^58,59^ In addition, the MOG and LG (extending to fusiform gyrus) are involved in perception of facial emotion expressions,^60^ while the precuneus is involved in autobiographical memory retrieval and integration with external emotional stimuli.^61–64^ The ACC is involved in emotion processing and sharing with others.^65,66^ During EAT, participants need to identify, understand, and feel the emotional states of the target who described his/her autobiographic events. Hence, our findings suggested that these regions may be involved in higher level of social cognition processing through integrating social stimuli with one’s own experiences to accurately empathize with others.^67,68^

Using ISC analyses, we identified a large network of synchronized activities among participants during EAT, with the strongest synchronized activations in bilateral STG. In EAT, participants processed the social information primarily through stories, thus the bilateral STG were engaged for voice perception and language comprehension.^69,70^ We also found strong ISC extending to higher-level cognitive processing networks, such as mentalizing network (e.g., the TPJ, precuneus)^58,59,71^ and mirror-neuron system (e.g., the IPL, IFG).^72^ These results are consistent with previous studies using ISC analyses during movie watching or narratives listening,^38,39,73,74^ suggesting ISC analyses offers a complementary perspective to traditional fMRI analyses.^32^

Interestingly, we found negative relationship between social anhedonia and the ISC of brain activation in left precentral gyrus, and further analysis showed that ISC of this region played a mediation role on the relationship between social anhedonia and affective empathy. The precentral gyrus is part of the mirror-neuron system,^75^ which gets involved in embodied process of empathy, for example, vicariously share other’s emotional states.^76^ Empirical evidence suggests that precentral gyrus is involved in empathic processing,^77,78^ and brain activities of precentral gyrus are related to self-report affective empathy.^79–81^ Hypoactivation of precentral gyrus was observed in schizophrenia patients during theory-of-mind (ToM) tasks.^82,83^ Besides, brain activities of precentral gyrus were negatively associated with negative symptoms in schizophrenia patients.^84^ Since we adopted the leave-one-out ISC analysis approach,^32,33^ lower ISC/ISDC could be interpreted as greater deviation from the normal pattern in the general population.^42^ Extending the previous findings, our study suggested that people with high levels of social anhedonia exhibited altered neural synchronization in precentral gyrus during empathic processing, which further undermined their affective empathy in social interaction.

Furthermore, we found a negative relationship between social anhedonia and the ISDC between the left STG and dorsal ACC (extending to dmPFC), which fully mediated the relationship of social anhedonia with affective empathy. The STG is recognized as a hub that conveys vocal information to other brain systems for subsequent cognitive or emotional processing.^85^ The dorsal ACC, on the other hand, is involved in emotion processing and affective-perceptual aspect of empathy.^66,86^ Previous studies suggested that schizophrenia patients showed diminished activation of dACC during pain empathy.^87^ People with negative schizotypy also exhibited reduced functional connectivity of dACC during emotion perception.^88^ The adjacent dmPFC is involved in mentalizing,^59,71,89^ and inferencing the traits about others.^75^ Previous study using naturalistic stimuli found a selective response in the dmPFC to movie scenes involving social interactions, which allowed participants to learn about the intentions and features of the characters.^90^ Thus, our findings suggested that people with social anhedonia would be less able to utilize verbal information for sharing as well as inferring the targets’ emotions during naturalistic social scenes, which may further impede their affective response to other’s emotions during social interaction. Although we found a positive relationship between cognitive empathy and the ISDC between the left STG and dorsal ACC, we did not further examine the mediation effect, because it was not strong enough to survive *Bonferroni* corrections. Further research on the relationship between cognitive empathy and neural synchronization is needed.

Positive schizotypy was negatively associated with the ISDC between the right PaCG and right LG. The PaCG is a key region in sensorimotor network, and the lingual gyrus is involved in processing external social stimuli, such as facial expressions.^60^ Hence, we assumed abnormal processing of target’s facial expressions during EAT in people with high levels of perceptual aberration. However, no association between such ISDC and self-report empathy was observed in our study. Therefore, it remained unclear whether and how this altered ISDC would affect social cognitive processing in people with positive schizotypy.

Notably, we found significant relationships of schizotypal traits with neural synchronization, but not with brain activations related to emotional distance. Similarly, another study employing both GLM and ISC analyses also found that schizotypal traits were closely linked to alterations in neural synchronization rather than neural activations.^43^ Previous studies have demonstrated high accuracy of the ISC analysis in identifying schizophrenia patients,^91^ and our findings further suggested the ISC approach as a sensitive method to detect individual differences related to schizotypy.^44^ On the other hand, it is plausible that altered social cognition in people with elevated levels of schizotypy may be attributable to the inability to synchronize neural activities with other people, thereby impeding mutual understanding and successful social interaction.^43,92,93^

Several limitations of this study should be mentioned. First, seeds for the ISDC analyses in this study were defined solely on basis of the results gathered from the GLM and ISC analyses in brain activation. Future study may take “theory-driven” brain regions that involved in processing of cognitive and affective empathy for ISDC analysis. Second, we used a college sample with varying degree of schizotypal features to demonstrate the correlations between neural synchronization during EAT and schizotypy. Future studies may recruit discrete groups of participants having high versus low levels of schizotypy to further explore distinct synchronization patterns between the two groups.^44^ Finally, we only measured positive and negative dimensions of schizotypy, and largely ignored the disorganized dimension of schizotypy.^9^ Future studies can use the Multidimensional Schizotypy Scale (MSS)^94,95^ to cover three dimensions of schizotypy.

In conclusion, our findings highlighted the reduced neural synchrony during empathic processing related to negative schizotypy, including synchronization of brain activation in the precentral gyrus, as well as dynamic connectivity between the STG and ACC, and their relationships with affective empathy. Our study may shed light on the neural mechanisms of social cognitive deficits in schizophrenia patients, and provide potential targets for intervention.

## Supplementary Material

Supplementary material is available at https://academic.oup.com/schizophreniabulletin/.

sbad182_suppl_Supplementary_Tables_S1-S3
