# Supplementary material for: Negative Schizotypy Associated With Weaker Intersubject Correlation in Dynamic Functional Connectivity During Empathic Accuracy Task
Source: Schizophr Bull. 2025 Mar 4;51(Suppl 2):S183–93. doi: 10.1093/schbul/sbad182 (PMC11879588; doi:10.1093/schbul/sbad182)
Supplement: sbad182_suppl_Supplementary_Tables_S1-S3 [file sbad182_suppl_supplementary_tables_s1-s3.docx]

**Supplementary Material**

1. ***The fMRI scanning and preprocessing***

A 3T GE Discovery MR750 scanner and an 8-channel head-coil were used for MRI data acquisition. T2-weighted echo-planer imaging (EPI) sequence was applied for functional MRI with the following parameters: repetition time (TR) = 2000ms, echo time (TE) = 30ms, flip angle = 90°. Thirty-seven slices in transverse plane were acquired in interleaved order (slice thickness = 3.5mm, matrix size = 64 × 64, field of view (FoV) = 220mm, voxel size = 3.4mm × 3.4mm × 3.5mm). T1-weighted MPRAGE sequence was used for anatomical image with the following parameters: TR = 6.65ms, TE = 2.93ms, filp angle = 12°, matrix size = 256 × 256, FoV = 256mm, slice thickness = 1mm, voxel size = 1mm × 1mm × 1mm. Structural images were examined by a qualified neuroradiologist to exclude any participant with structural abnormity.

Images were preprocessed using SPM12 (Wellcome Centre for Human Neuroimaging, University College London, UK, http://www.fil.ion.ucl.ac.uk/spm) and custom scripts in MATLAB 2014 (Mathworks, Natick, MA, USA). First, the T1-weighted structural image of each participant was segmented and normalized into Montreal Neurological Institute (MNI) space. Then, functional images were slice-timing corrected, realigned and coregistered to the skull-stripped T1 image for each participant. Framewise displacement (FD) was calculated in translation and rotation separately^51^ and those participants who had maximum FD greater than 1.5mm for translation or 1.5 degree for rotation were excluded from further analysis. The functional images were then normalized to MNI standard space, resampled to a voxel size of 3mm × 3mm × 3mm and smoothed using Gaussian kernel with 8mm full-width at half-maximum (FWHM).

1. **Descriptive statistics of Schizotypal Personality Questionnaire and the Associations of brain activation with dimensional schizotypy**

***The Schizotypal Personality Questionnaire***

The SPQ (Raine, 1991) is a valid self-report scale for measuring features of DSM-III-R schizotypal personality disorder with a three-factor model, including cognitive-perceptual, the interpersonal, and the disorganized factors. The revised Chinese version of SPQ has good reliability and validity (Chen et al., 1997).

**Table S1. Descriptive Statistics of the Schizotypal Personality Scale**

|  | **Entire Sample (*n* = 47)** | | | | | |
| --- | --- | --- | --- | --- | --- | --- |
|  | Mean | SD | Range | | Skewness | Kurtosis |
|  |  |  | Min | Max |  |  |
| SPQ_Cog_Per | 8.96 | 6.31 | 0 | 26 | 0.856 | 0.138 |
| SPQ_Int | 8.83 | 7.35 | 0 | 30 | 0.872 | 0.432 |
| SPQ_Diso | 4.62 | 4.45 | 0 | 16 | 1.001 | 0.101 |

*Note:* SPQ, Schizotypal Personality Questionnaire; Cog_Per, Cognitive-Perceptual dimension; Int, Interpersonal dimension; Diso, Disorganized dimension.

**Table S2. Results of the associations between ISDC, schizotypy, and self-report empathy**

| **ROI Labels** | **Predictors** | **Region** | **L/R** | **BA** | **Cluster Size** | **MNI** | | | ***t*** | ***p*** | **QCAE_CE** | | | **QCAE_AE** | | |
| --- | --- | --- | --- | --- | --- | --- | --- | --- | --- | --- | --- | --- | --- | --- | --- | --- |
|  |  |  |  |  |  | **x** | **y** | **z** |  |  | ***r*** | ***p*** | ***df*** | ***r*** | ***p*** | ***df*** |
| ROI1  (R.STG) | Cog_Per | NONE | | | | | | | | |  | | | | | |
|  | Int |  |  |  |  |  |  |  |  |  |  |  |  |  |  |  |
|  | Diso |  |  |  |  |  |  |  |  |  |  |  |  |  |  |  |
| ROI2  (L.STG) | Cog_Per |  |  |  |  |  |  |  |  |  |  |  |  |  |  |  |
|  | Int | Anterior cingulate cortex | L | 32 | 196 | 0 | 48 | 12 | -4.64 | <0.001 | 0.26 | 0.074 | 45 | 0.38 | 0.008 | 45 |
|  | Diso |  |  |  |  |  |  |  |  |  |  |  |  |  |  |  |
| ROI3  (L.MOG) | Cog_Per | NONE | | | | | | | | |  | | | | | |
|  | Int |  |  |  |  |  |  |  |  |  |  |  |  |  |  |  |
|  | Diso |  |  |  |  |  |  |  |  |  |  |  |  |  |  |  |
| ROI4  (R.MOG) | Cog_Per | NONE | | | | | | | | |  | | | | | |
|  | Int |  |  |  |  |  |  |  |  |  |  |  |  |  |  |  |
|  | Diso |  |  |  |  |  |  |  |  |  |  |  |  |  |  |  |
| ROI5  (L.PCU) | Cog_Per |  |  |  |  |  |  |  |  |  |  |  |  |  |  |  |
|  | Int |  |  |  |  |  |  |  |  |  |  |  |  |  |  |  |
|  | Diso | Anterior cingulate cortex | R | 32 | 85 | 12 | 33 | 21 | -3.43 | 0.040 | 0.11 | 0.450 | 45 | -0.19 | 0.191 | 45 |
| ROI6  (R.LG) | Cog_Per | NONE | | | | | | | | |  | | | | | |
|  | Int |  |  |  |  |  |  |  |  |  |  |  |  |  |  |  |
|  | Diso |  |  |  |  |  |  |  |  |  |  |  |  |  |  |  |
| ROI7  (R.PaCG) | Cog_Per | NONE | | | | | | | | |  | | | | | |
|  | Int |  |  |  |  |  |  |  |  |  |  |  |  |  |  |  |
|  | Diso |  |  |  |  |  |  |  |  |  |  |  |  |  |  |  |
|  |  |  | | | | | | | | |  | | | | | |
| ROI8  (R.ACC) | Cog_Per |  |  |  |  |  |  |  |  |  |  |  |  |  |  |  |
|  | Int | Inferior frontal gyrus | L | 9 | 76 | -39 | 15 | 27 | 5.00 | 0.029 | -0.30 | 0.038 | 45 | -0.04 | 0.784 | 45 |
|  |  | Cuneus | R | 18 | 76 | 18 | -81 | 33 | -4.52 | 0.029 | 0.19 | 0.194 | 45 | 0.12 | 0.436 | 45 |
|  |  | Superior temporal gyrus | L | 42 | 72 | -57 | -30 | 21 | -4.04 | 0.037 | 0.18 | 0.240 | 45 | -0.07 | 0.655 | 45 |
|  |  | Superior frontal gyrus | L | 6 | 78 | -9 | 18 | 51 | -3.94 | 0.025 | 0.28 | 0.240 | 45 | 0.01 | 0.945 | 45 |
|  | Diso |  |  |  |  |  |  |  |  |  |  |  |  |  |  |  |

*Note*: The table exhibits the results of the multiple regression analyses between ISDC and schizotypal traits measured by SPQ, while controlling for age and gender (left-side of the dotted line). Significant threshold was set at voxel-level uncorrected *p* < 0.005 and cluster-level family-wise error (FWE) corrected *p* < 0.05. Corresponding values of the significant clusters from the analyses with schizotypy were extracted for correlation analyses with self-report cognitive and affective empathy of the QCAE (right-side of the dotted line). No significant regions were identified in rows that are blank. ROI, region of interest; L, left; R, right; BA, Brodmann areas; MNI, Montreal Neurological Institute; Cog_Per, Cognitive Perceptual dimension of the SPQ; Int, Interpersonal dimension of the SPQ; Diso, Disorganized dimension of the SPQ; QCAE, Questionnaire of Cognitive and Affective Empathy; CE, Cognitive Empathy; AE, Affective Empathy; R.STG, right superior temporal gyrus; L.STG, left superior temporal gyrus; L.MOG, left middle occipital gyrus; R.MOG, right middle occipital gyrus; L.PCU, left precuneus; R.LG, right lingual gyrus; R.PaCG, right paracentral gyrus; R.ACC, right anterior cingulate cortex.

1. **Inter-subject correlation analyses for dynamic connectivity (ISDC)**

**Table S3. Results of the inter-subject correlation of dynamic connectivity (ISDC)**

| **ROI Labels** | **Region** | **L/R** | **BA** | **Cluster Size** | **MNI** | | | ***t*** | ***p*** |
| --- | --- | --- | --- | --- | --- | --- | --- | --- | --- |
|  |  |  |  |  | x | y | z |  |  |
| ROI1  (R.STG) | Superior temporal gyrus | L | 22 | 859 | -57 | -15 | 0 | 12.53 | <0.001 |
|  | Superior temporal gyrus | R | 22 | 883 | 63 | -12 | 3 | 12.37 | <0.001 |
|  | Middle occipital gyrus | L | 18 | 324 | 30 | -93 | 0 | 8.31 | <0.001 |
|  | Middle occipital gyrus | R | 18 | 1527 | -30 | -93 | 0 | 7.67 | <0.001 |
|  | Precuneus | L | 7 | 417 | -15 | -36 | 48 | 6.28 | <0.001 |
|  | Middle frontal gyrus | L | 6 | 37 | -45 | 6 | 51 | 5.64 | 0.029 |
|  | Precentral gyrus | R | 4 | 73 | 42 | -12 | 54 | 5.49 | 0.001 |
|  | Medial frontal gyrus | L | 8 | 154 | -3 | 33 | 42 | 5.36 | <0.001 |
|  | Middle frontal gyrus | R | 6 | 154 | 39 | 12 | 48 | 5.22 | <0.001 |
|  | Anterior cingulate gyrus | L | 24 | 242 | -6 | 36 | 9 | 5.20 | <0.001 |
|  | Inferior frontal gyrus | L | 47 | 35 | -45 | 27 | -9 | 5.08 | 0.036 |
|  | Inferior frontal gyrus | R | 47 | 126 | 48 | 27 | -6 | 4.79 | <0.001 |
|  | Precentral gyrus | L | 9 | 60 | -39 | 9 | 30 | 4.78 | 0.003 |
| ROI2  (L.STG) | Superior temporal gyrus | R | 41 | 911 | 63 | -18 | 3 | 12.76 | <0.001 |
|  | Superior temporal gyrus | L | 41 | 726 | -51 | -24 | 3 | 12.16 | <0.001 |
|  | Middle occipital gyrus | R | 18 | 2749 | 30 | -93 | 0 | 9.21 | <0.001 |
|  | Middle occipital gyrus | L | 18 | 208 | -30 | -93 | 0 | 7.81 | <0.001 |
|  | Paracentral lobule | L | 5 | 783 | -15 | -36 | 48 | 6.47 | <0.001 |
|  | Inferior frontal gyrus | L | 47 | 66 | -45 | 27 | -9 | 6.47 | 0.002 |
|  | Middle frontal gyrus | L | 6 | 43 | -45 | 6 | 51 | 6.13 | 0.017 |
|  | Medial frontal gyrus | L | 8 | 706 | -3 | 33 | 45 | 5.94 | <0.001 |
|  | Middle frontal gyrus | R | 6 | 385 | 36 | 9 | 48 | 5.83 | <0.001 |
|  | Precentral gyrus | R | 4 | 44 | 45 | -12 | 51 | 5.16 | 0.016 |
|  | Supramarginal gyrus | R | 40 | 64 | 54 | -33 | 45 | 5.16 | 0.002 |
|  | Middle frontal gyrus | L | 10 | 53 | -36 | 51 | 0 | 5.10 | 0.007 |
|  | Middle frontal gyrus | L | 9 | 68 | -33 | 18 | 30 | 4.82 | 0.002 |
|  | Postcentral gyrus | R | 2 | 41 | 60 | -15 | 30 | 4.58 | 0.021 |
|  | Putamen | L |  | 41 | -27 | 9 | 6 | 4.45 | 0.021 |
| ROI3  (L.MOG) | Superior temporal gyrus | R | 41 | 551 | 57 | -21 | 9 | 6.82 | <0.001 |
|  | Middle occipital gyrus | L | 19 | 738 | -30 | -75 | 21 | 6.44 | <0.001 |
|  | Superior temporal gyrus | L | 22 | 436 | -51 | -12 | -3 | 6.06 | <0.001 |
|  | Lingual gyrus | L | 18 | 33 | -12 | -72 | -9 | 5.40 | 0.032 |
|  | Precuneus | L | 7 | 96 | -12 | -60 | 45 | 4.64 | <0.001 |
|  | Supplementary Motor Area | L | 6 | 35 | -6 | 9 | 60 | 4.43 | 0.025 |
|  | Inferior frontal gyrus | R | 47 | 42 | 51 | 27 | 0 | 4.19 | 0.011 |
| ROI4  (R.MOG) | Middle occipital gyrus | L | 18 | 398 | -15 | -87 | 21 | 6.41 | <0.001 |
|  | Superior temporal gyrus | R | 41 | 129 | 57 | -24 | 6 | 5.28 | <0.001 |
|  | Middle temporal gyrus | L | 22 | 58 | -63 | -33 | 3 | 4.05 | 0.001 |
|  | Precuneus | L | 7 | 29 | -3 | -51 | 48 | 3.83 |  |
| ROI5  (L.PCU) | Superior temporal gyrus | L | 42 | 434 | -66 | -21 | 12 | 5.80 | <0.001 |
|  | Orbital frontal gyrus | R | 46 | 161 | 42 | 45 | -3 | 5.29 | <0.001 |
|  | Superior temporal gyrus | R | 22 | 267 | 60 | 3 | 0 | 5.17 | <0.001 |
|  | Angular gyrus | R | 39 | 89 | 48 | -60 | 33 | 5.10 | <0.001 |
|  | Middle occipital gyrus | L | 17 | 92 | -21 | -93 | 6 | 5.01 | <0.001 |
|  | Superior frontal gyrus | L | 10 | 175 | -36 | 57 | 0 | 4.85 | <0.001 |
|  | Inferior parietal lobe | R | 40 | 89 | 60 | -30 | 45 | 4.75 | <0.001 |
|  | Superior frontal gyrus | L | 6 | 148 | -6 | 15 | 66 | 4.61 | <0.001 |
|  | Lingual gyrus | R | 18 | 71 | 21 | -90 | -9 | 4.46 | 0.001 |
|  | Inferior frontal gyrus | L | 44 | 64 | -45 | 15 | 0 | 4.44 | 0.003 |
|  | Inferior parietal lobule | L | 40 | 34 | -48 | -45 | 48 | 4.33 | 0.047 |
|  | Superior parietal lobe | R | 7 | 34 | 15 | -51 | 57 | 4.29 | 0.047 |
| ROI6  (R.LG) | Superior temporal gyrus | R | 22 | 57 | 57 | -15 | 3 | 4.65 | 0.001 |
|  | Lingual gyrus | R | 18 | 50 | 12 | -72 | 0 | 4.31 | 0.003 |
|  | Middle temporal gyrus | R | 22 | 50 | 57 | -39 | 3 | 4.19 | 0.003 |
| ROI7  (R.PaCG) | Precuneus | R | 7 | 44 | 3 | -60 | 36 | 4.97 | 0.008 |
|  | Anterior cingulate gyrus | L | 32 | 74 | -3 | 42 | -6 | 4.77 | <0.001 |
| ROI8  (R.ACC) | Inferior parietal lobule | R | 40 | 189 | 57 | -39 | 45 | 6.56 | <0.001 |
|  | Middle frontal gyrus | L | 10 | 82 | -39 | 54 | 6 | 6.01 | <0.001 |
|  | Superior temporal gyrus | L | 22 | 158 | -57 | -6 | -9 | 5.51 | <0.001 |
|  | Middle frontal gyrus | R | 10 | 103 | 45 | 48 | 15 | 5.20 | <0.001 |
|  | Superior temporal gyrus | R | 21 | 144 | 66 | -15 | -6 | 4.90 | <0.001 |
|  | Medial frontal gyrus | L | 32 | 76 | -3 | 30 | 33 | 4.81 | <0.001 |
|  | Cingulate gyrus | L | 31 | 60 | -3 | -30 | 42 | 4.63 | 0.001 |
|  | Superior temporal gyrus | L | 22 | 61 | -57 | -54 | 9 | 4.57 | 0.001 |
|  | Middle frontal gyrus | R | 8 | 33 | 27 | 33 | 36 | 4.41 | 0.027 |
|  | Cuneus | L | 17 | 29 | 0 | -87 | 12 | 4.11 | 0.044 |

*Note*: Significant threshold was set at voxel-level uncorrected *p* < 0.001 and cluster-level family-wise error (FWE) corrected *p* < 0.05. ROI, region of interest; L, left; R, right; BA, Brodmann areas; MNI, Montreal Neurological Institute; R.STG, right superior temporal gyrus; L.STG, left superior temporal gyrus; L.MOG, left middle occipital gyrus; R.MOG, right middle occipital gyrus; L.PCU, left precuneus; R.LG, right lingual gyrus; R.PaCG, right paracentral gyrus; R.ACC, right anterior cingulate cortex.
